# Sonographic and Biochemical Markers in Gestational Diabetes Mellitus: A Prospective Cohort Study at a Tertiary Hospital in Greece

**DOI:** 10.7759/cureus.90381

**Published:** 2025-08-18

**Authors:** Paraskevas Anagnostopoulos, Angeliki Bolou, Vikentia Harizopoulou, Paraskevi Giaxi, Panagiota Tzela, Maria Papastamatiou, Thomas Vrekoussis, Kleanthi Gourounti

**Affiliations:** 1 Department of Obstetrics and Gynaecology, University of Crete, Heraklion, GRC; 2 Department of Midwifery, University of West Attica, Athens, GRC

**Keywords:** gestational diabetes mellitus, papp-a, polyhydramnios, uterine artery pulsatility index, β-hcg

## Abstract

Objective

The present study investigates biochemical and sonographic markers in pregnant women with gestational diabetes mellitus (GDM), aiming to identify correlations related to demographic factors (maternal age and previous pregnancies), disease severity, and differences among pregnant women with GDM based on treatment (diet or insulin administration).

Methods

This study includes 16 cases of GDM with singleton pregnancies, who attended the outpatient clinic of the University General Hospital of Heraklion, Heraklion, Greece, and had no comorbidities. The control group consisted of 25 healthy pregnant women who visited the same outpatient clinics during the same period. Personal data were collected through anonymous questionnaires regarding age, number of previous pregnancies, and any existing conditions. In cases of GDM, women were categorized based on whether they were treated with insulin or managed with a carbohydrate-restricted diet. Biochemical markers - pregnancy-associated plasma protein A (PAPP-A) and β-human chorionic gonadotrophin (β-hCG) - were measured. These levels were converted to MoM (multiples of the normal median) values, allowing direct comparison with expected values for gestational age. Ultrasonographic indices - uterine artery pulsatility index (Ut-A PI) and large for gestational age (LGA) - were assessed using the ultrasound Voluson 730 (General Electric, Boston, MA, USA) device in the Gynecology-Obstetrics Department.

Results

Our results showed a trend for reduced β-hCG levels in the GDM group compared to the control group, but these levels tended to be higher in the GDM subgroup treated with insulin. Ut-A PI was reduced in the GDM subgroup with insulin, and there were more LGA fetuses. Age was positively associated with β-hCG in the diet group. The same group had PAPP-A positively associated with age and Ut-A PI in a linear fashion, and negatively associated with the number of previous pregnancies. In the insulin-treated GDM group, PAPP-A was positively correlated with Ut-A PI (in a linear fashion), LGA, and negatively with β-hCG.

Conclusion

In this article, we recommend early, universal screening of all pregnant women during the early weeks of the first trimester. Biochemical markers, in association with fasting plasma glucose levels, would predict the risk of gestational diabetes in the pregnant woman. We suggest early testing and intervention to prevent the development of fetal hyperinsulinemia and reduce the likelihood of metabolic disease later in life.

## Introduction

Gestational diabetes mellitus (GDM) is a condition involving disturbances in carbohydrate, fat, and protein metabolism, first identified during pregnancy, without pre-existing diabetes. All pregnant women should be assessed for gestational diabetes at 24-28 weeks of gestation [1,2]. Pregnant women with GDM may have high blood glucose levels before being diagnosed at 24 weeks, potentially affecting fetal growth due to maternal hyperglycemia. Using first-trimester maternal serum biomarkers can lead to early diagnosis and intervention for GDM, improving maternal and fetal health [3].

O'Sullivan and Mahan first described the condition in 1964 as a deficiency or reduced activity of insulin in peripheral tissues [4]. The main manifestation of the disorder is elevated maternal blood glucose levels.

Globally, GDM complicates approximately 15% of pregnancies. In Europe, the prevalence was recently reported to be 8%, depending on the population of each geographic area studied [2]. The rising incidence of this condition over the last 15 years is concerning and is linked to increasing maternal age and obesity rates among Greek women as well.

Women with GDM are at increased risk of short-term and long-term complications, such as the development of preeclampsia and the establishment of hyperglycemia as type II diabetes mellitus (T2DM). Additionally, neonates of diabetic mothers are at risk of complications such as macrosomia, which leads to an increased risk of birth trauma, as well as a higher likelihood of developing metabolic disease later in life [5].

It is essential to recognize that this condition can be effectively managed through the collaboration of specialists, including obstetricians, nutritionists, and endocrinologists. Such interdisciplinary cooperation ensures the well-being of both the mother and the infant. The management of GDM includes an individualized nutritional program low in carbohydrates, alongside the initiation of physical exercise. It is particularly important to effectively manage the pregnant woman's body weight, along with monitoring glucose levels, to achieve the necessary glycemic control [6].

## Materials and methods

Study design

This study involved 16 pregnant women with singleton pregnancies and GDM, monitored at the outpatient clinic of the University General Hospital of Heraklion, Heraklion, Greece. None of the participants had comorbidities. Twenty-five healthy pregnant women were recruited as controls. The study was approved by the Research Ethics Committee (Ref. No. 14853/02-05-2025), and all participants provided informed consent.

Personal data were collected using a standardized medical history form in the form of an anonymous questionnaire filled out by each participant. Age, number of previous pregnancies, and the presence of any pre-existing medical conditions were recorded. Women in the GDM group were assigned to two groups according to their treatment method: insulin therapy or dietary management with carbohydrate restriction.

The Biochemistry Department of the University General Hospital of Heraklion analyzed biochemical markers pregnancy-associated plasma protein A (PAPP-A) and β-human chorionic gonadotrophin (β-hCG) as part of routine monitoring. The concentrations of PAPP-A and β-hCG were transformed into multiples of the median (MoM), allowing for standardized comparisons across different gestational ages by normalizing the measurements to the anticipated median values.

Ultrasound markers, including uterine artery pulsatility index (Ut-A PI) and large for gestational age (LGA), were assessed during second-trimester ultrasounds using a Voluson 730 ultrasound system (General Electric, Boston, MA, USA), installed in the Department of Obstetrics and Gynecology at the University General Hospital of Heraklion.

Statistical analysis

Statistical analysis was performed using GraphPad Prism version 8.0 software (GraphPad Software, Inc., La Jolla, CA, USA). Student’s t-test was used to assess statistically significant differences between groups. Pearson’s correlation coefficient (r) was utilized for continuous variables, whereas Spearman’s rank correlation coefficient (ρ, rho) was employed for ordinal variables or in analyses involving both continuous and ordinal variables, such as heatmap matrix analyses. The interpretation of absolute values for Pearson’s r and Spearman’s rho followed these thresholds: |r| = 0.01-0.19, negligible or very weak correlation; |r| = 0.20-0.29, weak correlation; |r| = 0.30-0.39, moderate correlation; |r| = 0.40-0.69, strong correlation; and |r| ≥ 0.70, very strong correlation. Error bars in graphical representations indicate the standard error of the mean (SEM), unless otherwise specified. For figures characterizing each sample group, the standard deviation (SD) was used instead. Each figure includes a specific legend detailing the statistical measures presented.

## Results

Initially, the characteristics of the two groups were studied, and no distinction was made between those who received insulin for the management of GDM and those who followed a diet with reduced carbohydrate intake only. The mean age of the pregnant women with GDM was 31.88 ± 6.89, and they had a mean number of pregnancies of 2.063 ± 1.28, including the pregnancy during which they presented to the outpatient clinics of the University General Hospital of Heraklion. Similarly, for the control group, 25 healthy pregnant women who visited the outpatient clinics during the same period were selected. The mean age in this group was 28.83 ± 5, and they had a mean number of pregnancies of 1.864 ± 1.03.

Subsequently, a comparison was made of the levels of the biochemical markers β-hCG and PAPP-A, which were measured from serum samples collected during the pregnant women's visit to the hospital. These two proteins are among the most commonly used markers in everyday clinical practice for monitoring the progression of pregnancy. In the statistical analysis that followed, comparing the levels in the GDM group to those of the control group, a difference was found in β-hCG: 1.11 ± 0.14 MoM vs. 1.331 ± 0.27 MoM, while PAPP-A levels showed no significant difference: 1.2 ± 0.1874 MoM vs. 1.268 ± 0.12 MoM, respectively (Table 1).

**Table 1:** Comparison of GDM group and control group GDM: gestational diabetes mellitus; PAPP-A: pregnancy-associated plasma protein-A; β-hCG: β-human chorionic gonadotrophin; MoM: multiples of the median

Parameter	GDM Group (n = 16)	Control Group (n = 25)
Age (years)	31.88 ± 6.89	28.83 ± 5
No. of pregnancies	2.063 ± 1.28	1.864 ± 1.03
PAPP-A (MoM)	1.2 ± 0.18	1.268 ± 0.12
β-hCG (MoM)	1.11 ± 0.14	1.331 ± 0.27

The women recruiting the GDM group were divided into diet-controlled (n = 8) and insulin-treated (n = 8) subgroups. Table 2 shows that there were no differences in demographic characteristics and basic biochemical markers between the two groups.

**Table 2:** Comparison of diet-treated GDM group and insulin-treated GDM group GDM: gestational diabetes mellitus; PAPP-A: pregnancy-associated plasma protein-A; β-hCG: β-human chorionic gonadotropin; MoM: multiples of the median

Parameter	Diet-Treated Group (n = 8)	Insulin-Treated Group (n = 8)	p-value
Age (years)	33.25 ± 8.86	30.50 ± 4.38	0.44
No. of pregnancies	2.4 ± 1.6	1.8 ± 0.9	0.35
PAPP-A (ΜοΜ)	1.01 ± 0.15	1.30 ± 0.35	0.6
β-hCG (ΜοΜ)	0.99 ± 0.21	1.23 ± 0.21	0.42

Ultrasonographic indices among GDM groups

**Figure 1:**
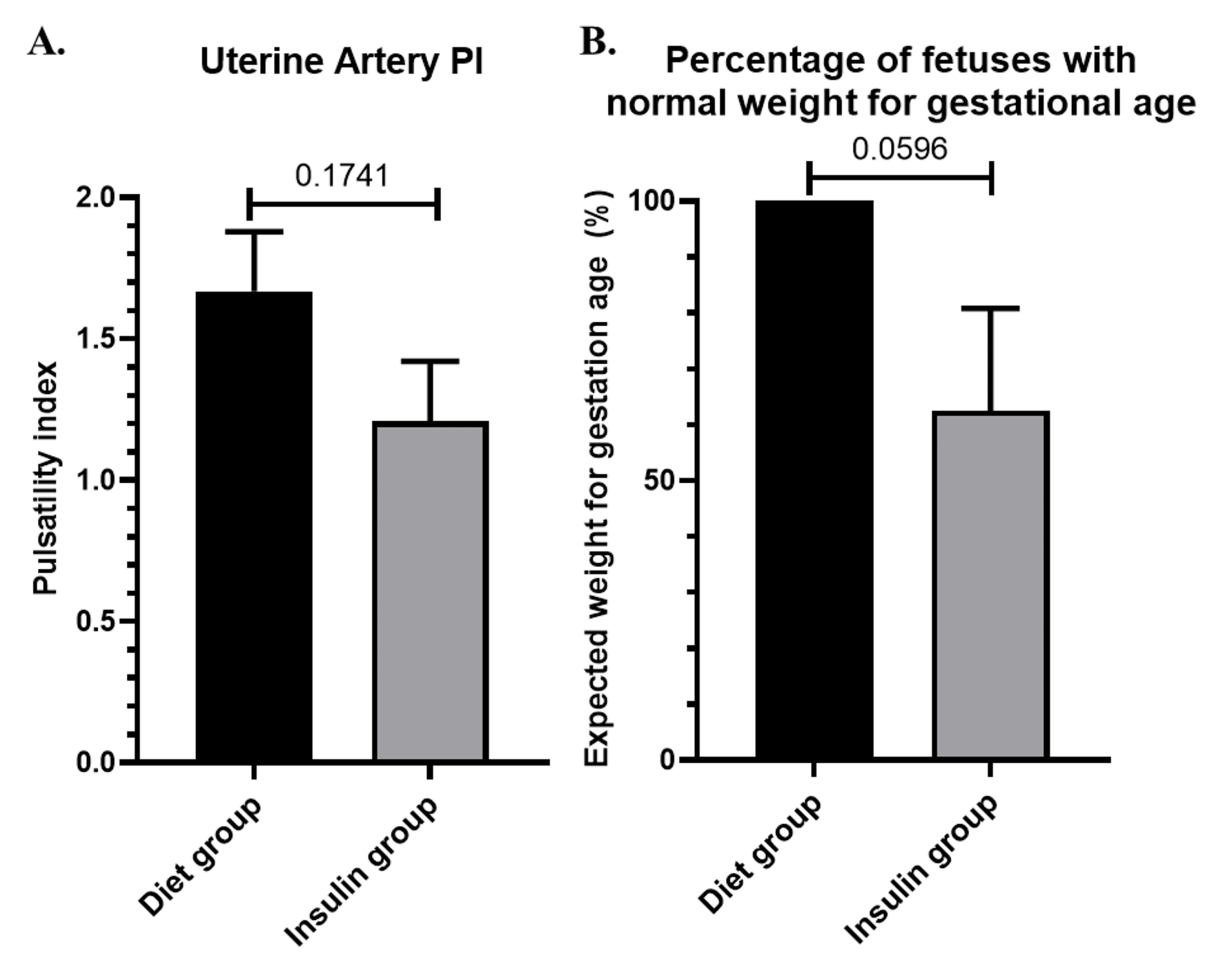
Levels of ultrasound markers (A) Uterine artery PI; (B) fetal size for gestational age, expressed as the percentage of fetuses with normal weight. Results are presented as mean ± SEM. PI: pulsatility index; SEM: standard error of the mean

Second-trimester ultrasound assessed Ut-A PI and fetal size indicators (Figure 1A). Regarding the Ut-A PI, the diet-treated group did not differ (p = 0.1741) from the insulin-treated group (1.668 ± 0.2107 vs. 1.208 ± 0.2126). Furthermore, a decreased proportion of insulin-treated group fetuses had normal weight compared to the diet-treated group (62.50% ± 18.30 vs. 100.0% ± 0.0) (Figure 1B). In other words, there was a tendency (p = 0.0596) for LGA fetuses (>90th percentile) in the insulin GDM group.

Correlation analyses

Spearman Correlation

In the diet-controlled GDM subgroup, PAPP-A was positively correlated with maternal age and Ut-A PI, and negatively correlated with previous pregnancies. In the insulin-treated subgroup, PAPP-A was positively correlated with Ut-A PI and LGA, and negatively correlated with β-hCG. Additionally, the Ut-A PI showed a negative correlation with both maternal age and number of pregnancies, and a positive correlation with LGA.

Spearman Correlation Analysis

Spearman correlation was used to examine the relationships between variables in each GDM treatment group, comparing ordinal values (age, number of pregnancies, and LGA status), and continuous values (PAPP-A, β-hCG, and Ut-A PI).

**Figure 2:**
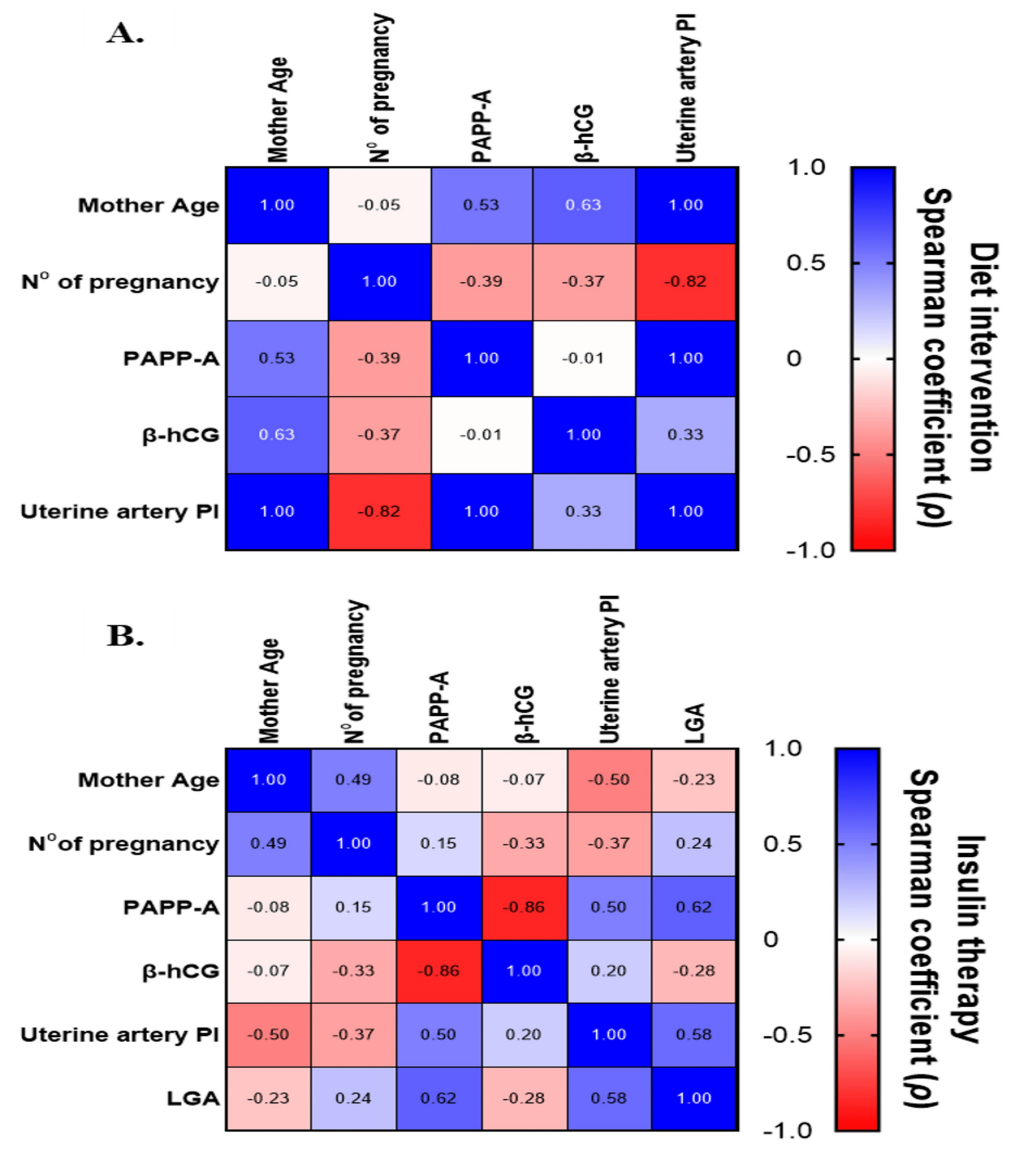
Heatmap comparing the Spearman correlation coefficient (ρ) values Heatmap comparing the Spearman correlation coefficient (ρ) values for the markers investigated in the group of pregnant women with GDM who received (A) dietary intervention and (B) insulin therapy. Positive (blue) or negative (red) values indicate positive or negative correlations, respectively, between the markers under investigation. GDM: gestational diabetes mellitus; PI: pulsatility index; β-hCG: β-human chorionic gonadotropin; PAPP-A: pregnancy-associated plasma protein-A; LGA: large for gestational age

Figure 2A shows no significant associations between age and number of pregnancies, or between PAPP-A and β-hCG in the diet-controlled group. Moderate correlations were seen between β-hCG and PI (ρ = 0.33), and between the number of pregnancies and both PAPP-A (ρ = -0.39) and β-hCG (ρ = -0.37). Strong positive correlations emerged between age and PAPP-A (ρ = 0.53) and between age and β-hCG (ρ = 0.63). Ut-A PI had a strong negative correlation with the number of pregnancies (ρ = -0.82), and very strong positive correlations with both age (ρ = 1.00) and PAPP-A (ρ = 1.00), with borderline statistical significance (p = 0.083).

In the insulin-treated group (Figure 2B), most correlations were weak or moderate. Notably, the Ut-A PI showed positive correlations with PAPP-A (ρ = 0.50) and LGA (ρ = 0.58), while being negatively correlated with maternal age (ρ = -0.50). A statistically significant and very strong negative correlation was found between PAPP-A and β-hCG (ρ = -0.86, p = 0.011), indicating a monotonic inverse relationship in this subgroup.

Pearson’s Correlation Analysis

**Figure 3:**
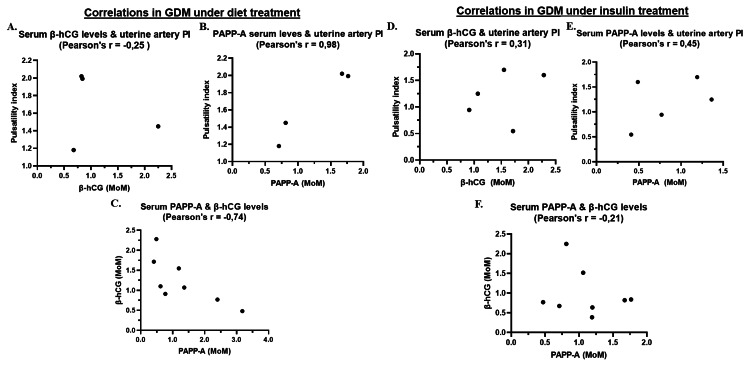
Dual-axis comparison plots of continuous markers investigated in the group of pregnant women with GDM who received (A-C) dietary intervention or (D-F) insulin therapy For each plot, the Pearson correlation coefficient (r) has been calculated. (A-C) Comparison of values in the diet group: (A) uterine artery PI and serum β-hCG levels, (B) uterine artery PI and serum PAPP-A levels, and (C) serum PAPP-A and β-hCG levels. (D-F) Comparison of values in the insulin group: (D) uterine artery PI and serum β-hCG levels, (E) uterine artery PI and serum PAPP-A levels, and (F) serum PAPP-A and β-hCG levels. GDM: gestational diabetes mellitus; PI: pulsatility index; β-hCG: β-human chorionic gonadotropin; PAPP-A: pregnancy-associated plasma protein-A

In the diet-controlled GDM subgroup (Figures 3A-3C), there was a weak negative linear correlation between β-hCG and Ut-A PI (r = -0.25, p = 0.7545), and between PAPP-A and PI (r = -0.21, p = 0.6111). However, PAPP-A showed an extraordinarily strong positive correlation with Ut-A PI (r = 0.98, p = 0.0230).

In the insulin-treated subgroup (Figures 3D-3F), β-hCG showed a moderate positive correlation with Ut-A PI (r = 0.31, p = 0.6081), while PAPP-A was strongly positively correlated with PI (r = 0.45, p = 0.4476). Notably, a strong negative correlation was found between PAPP-A and β-hCG (r = -0.74, p = 0.0369).

## Discussion

The regulation of maternal glucose levels changes during the onset and progression of pregnancy, as it must provide essential nutrients not only to the mother but also to the developing fetus. This is achieved through changes in glucose regulation caused by insulin resistance. Elevated serum glucose levels in pregnant women, who are diagnosed for the first time during pregnancy, characterize GDM. GDM-induced hyperglycemia can lead to complications for both mother and fetus [7,8].

The aim of the present study was to investigate whether some classic biochemical and ultrasound markers can predict the development of GDM or even its severity. Among the most common markers for assessing the course of a pregnancy is the measurement of serum β-hCG levels in pregnant women. Deviations from normal β-hCG levels have been directly linked to a multitude of pregnancy complications. For this reason, several studies have attempted to link β-hCG with GDM [9]. In most of these studies, results indicate that pregnant women with GDM have lower serum levels of the protein compared to the control group. Similar results were observed in our study, where a tendency for reduced β-hCG levels was noted in the GDM group [10,11].

A potential mechanism may relate to a latent phase like prediabetes, where women, despite having normal serum glucose levels, simultaneously experience hyperinsulinemia. Hyperinsulinemia reduces insulin growth factor binding protein 1 (IGFBP-1) and glycodelin levels, affecting trophoblast proliferation and potentially hCG production. The tendency we observed for increased β-hCG at the beginning of pregnancy in the GDM subgroup that later received insulin might reflect more severe insulin resistance - and thus less impact on the trophoblast - compared to the subgroup that underwent dietary intervention. It is plausible that this reflects a different compensatory mechanism at the placental level. Additionally, the positive correlation of β-hCG with age in the subgroup that made only dietary changes, which was not observed in women who received insulin, could be explained by a potentially limited capacity of the trophoblast for hCG production due to dysregulated insulin [12].

PAPP-A is a biochemical marker often measured early in pregnancy to predict complications. Reduced serum PAPP-A levels decrease the availability of insulin growth factor 1 (IGF-1). This reduction subsequently leads to increased insulin levels, disturbances in glucose metabolism, and insulin resistance. In our study, there was no apparent difference in PAPP-A levels between women with GDM and healthy pregnant women. Additionally, low PAPP-A levels at the beginning of pregnancy have been associated with an increased likelihood of insulin administration for GDM management. This was not confirmed in our study, as there were no significant differences in PAPP-A levels between the women with GDM who managed it with diet and those who received insulin [13,14].

PAPP-A levels during the first trimester of pregnancy can be used for GDM screening in combination with other markers. Therefore, in the context of our study, we attempted to identify such correlations that could potentially be used together to predict not only the development of GDM but also its severity. While statistical correlations were observed, further studies are required to assess whether these findings are clinically actionable. Using the Spearman correlation coefficient to analyze all the data collected in this study, we found that PAPP-A can correlate with various indicators, whether demographic, biochemical, or ultrasound [15]. Specifically, in the subgroup of patients who made dietary changes, PAPP-A levels were positively correlated with age and Ut-A PI, while there was a negative correlation with the number of pregnancies. In patients receiving insulin, PAPP-A levels positively correlated with Ut-A PI. No other significant correlations were found. Conversely, PAPP-A was found to have a positive association with LGA (not considered in the other subgroup due to the absence of an LGA fetus) and a negative association with β-hCG. All three of these correlations were strong [16,17].

Subsequently, using the Pearson correlation coefficient, we cross-evaluated the results produced using the Spearman correlation coefficient and investigated whether there is a linear correlation between the continuous markers used. In both GDM subgroups, there was a positive linear correlation of PAPP-A with the Ut-A PI, which was also observed in a previous study showing the simultaneous reduction of PAPP-A and Ut-A PI. In our study, this correlation was stronger in the diet subgroup. Additionally, PAPP-A showed a significant negative linear correlation with β-hCG in the insulin-treated subgroup [18].

The development of GDM, and even pre-existing uncontrolled diabetes, can lead to changes in the vascular endothelium, including the uterine artery. This is primarily due to inflammation and oxidative stress caused by high glucose levels in circulation. Therefore, the Ut-A PI may serve as a valuable tool not only for evaluating placentation and fetal development but also for the early diagnosis of GDM. Reduced PI may be linked to GDM development, according to some sources. Our study found it to be a significant marker, showing a clear decrease in the insulin subgroup. Additionally, using the Spearman correlation coefficient, beyond the relationships with β-hCG and PAPP-A mentioned, the Ut-A PI in both GDM subgroups seemed to decrease as the number of previous pregnancies increased, and this negative relationship was more significant in the dietary intervention subgroup. In the insulin subgroup, the Ut-A PI was more associated with age (negatively) and LGA (positively). According to studies, the Ut-A PI tends to increase with age, thus indicating a positive correlation. The observation of the opposite here might relate to GDM and is certainly interesting. The observation of the opposite in this context may suggest a GDM-related disruption of vascular adaptation [19].

## Conclusions

In conclusion, despite the small sample size, which is the main limitation of this study, part of the trends shown by our results align with international literature. One such example is the tendency for reduced β-hCG levels in the group that will develop GDM. Additionally, PAPP-A did not appear to differ significantly in the group that developed GDM but was strongly, positively correlated - even linearly - with the Ut-A PI, while there was a simple correlation with LGA. Our study indicates that combining biochemical and ultrasound markers with demographic data of pregnant women can predict not only preeclampsia but also the occurrence and severity of GDM, aiding in determining the therapeutic approach. 
